# Framework for life cycle assessment of livestock production systems to account for the nutritional quality of final products

**DOI:** 10.1002/fes3.143

**Published:** 2018-08-01

**Authors:** Graham A. McAuliffe, Taro Takahashi, Michael R. F. Lee

**Affiliations:** ^1^ Rothamsted Research Okehampton Devon UK; ^2^ University of Bristol Lanford Somerset UK

**Keywords:** environmental footprints, farm management, human nutrition, nutrient index, omega‐3, sustainable agriculture

## Abstract

Life cycle assessment (LCA) is widely regarded as a useful tool for comparing the environmental impacts of multiple livestock production systems. While LCA results are typically communicated in the form of environmental burdens per mass unit of the end product, it is increasingly becoming recognized that the product quality also needs to be accounted for to truly understand the value of a farming system to society. To date, a number of studies have examined environmental consequences of different food consumption patterns at the diet level; however, few have addressed nutritional variations of a single commodity attributable to production systems, leaving limited insight into how on‐farm practices can be improved to better balance environment and human nutrition. Using data from seven livestock production systems encompassing cattle, sheep, pigs, and poultry, this paper proposes a novel framework to incorporate nutritional value of meat products into livestock LCA. The results of quantitative case studies demonstrate that relative emissions intensities associated with different systems can be dramatically altered when the nutrient content of meat replaces the mass of meat as the functional unit, with cattle systems outperforming pig and poultry systems in some cases. This finding suggests that the performance of livestock systems should be evaluated under a whole supply chain approach, whereby end products originating from different farm management strategies are treated as competing but separate commodities.

## 1 INTRODUCTION

With increasing concern regarding environmental consequences of agricultural production worldwide, the importance of farming system evaluation has never been greater (Eisler et al., 2014; Gerber et al., 2013; Horton, Koh, & Guang, 2016). Among the plethora of evaluation methods, life cycle assessment (LCA) across agri‐food supply chains is considered to be one of the most informative tools to quantitatively compare environmental performances of multiple farming strategies at the systems level (de Vries & de Boer, 2010). Studies employing agri‐food LCA typically estimate pollution–production ratios as their primary outputs, for example kg CO_2_‐eq per unit of food produced, whereby systems represented by lower scores are judged to be socially more desirable. In the context of livestock production systems, the denominator depicting the quantity of production, or the functional unit, generally takes the form of output mass, such as 1 kg of liveweight, cold carcass weight, or deboned meat (McAuliffe, Chapman, & Sage, 2016; McAuliffe, Takahashi, Mogensen et al., 2017; de Vries, van Middelaar, & de Boer, 2015).

While this mass‐based approach provides a useful means of intercomparisons between different farming systems (McAuliffe, Takahashi, Orr, Harris, & Lee, 2018), the resultant indicators are not a holistic representation of the real function of the final product, in this case meat as a source of human nutrition. Recent research has begun to address this issue in the context of dietary comparisons, primarily focusing on the consumption side of agrifood systems (Hallström, Carlsson‐Kanyama, & Börjesson, 2015; Sonesson, Davis, Flysjö, Gustavsson, & Witthöft, 2017); Coelho, Pernollet, and van der Werf (2016), for example, examined the environmental impacts of hypothetical human diets with elevated omega‐3 polyunsaturated fatty acid (PUFA) intake, which is technologically possible by adjusting livestock feeds to promote a higher omega‐3 content in animal tissues. Society‐wide dietary shifts, however, require drastic changes in supply chain structure as well as consumers’ opinions, and therefore can only be achieved over a long period of time (Smil, 2000). In addition, as any human diet is composed of a large number of food groups originating from multiple farms, implications of these studies on agricultural systems producing each commodity are not immediately clear. The latter problem is further exacerbated by the fact that a change in farming methods, however minor, often disrupts the flow of nutrients within the production environment and consequently leads to knock‐on effects on chemical compositions of the end products, and ultimately their nutritional value to humans. This, in turn, poses a question about the implicit assumption behind the majority of dietary comparison studies (and others adopting mass‐based functional units) that all products are qualitatively homogenous. In order to draw short to medium‐term recommendations for commercial agricultural producers to improve their environmental performance, it is therefore necessary to establish an LCA methodology that can account for nutritional compositions of individual food groups that are produced under multiple production systems.

Using omega‐3 content of meat products as a starting example, this paper aims to demonstrate the effect of incorporating product quality, as opposed to quantity, into the carbon footprinting framework for a range of meat products. Meat consumption, particularly that of red and processed meat, is commonly associated with an increased risk of cardiovascular disease (CVD) (Daviglus, Pirzada, & He, 2017). With red meat being low (typically <5%) in total fat, the causality appears to be driven by high proportions of short chain saturated fatty acids (SFA), particularly C12:0 (lauric acid), C14:0 (myristic acid) and C16:0 (palmitic acid; Micha & Mozaffarian, 2010), together with ω‐6:ω‐3 (omega‐6:omega‐3) ratios as high as 15:1 (Warren et al., 2008a). This, in turn, is perceived to be contributing to “unhealthy” Western diets with typical ω‐6:ω‐3 ratios in excess of 12:1, while the medically recommended ratio is around 3:1 (Simopoulos, 2006). A growing body of studies indicate, however, that advice on dietary restrictions of lean red meat may, in fact, be counterproductive to prevention of noncommunicable disease (Binnie, Barlow, Johnson, & Harrison, 2014). C18:0 (stearic acid), for instance, has a neutral effect on low‐density lipoprotein cholesterol levels, with no clear indication of differences in health benefits or risks between different livestock products (Grundy, 1994; Schneider, Cowles, Stuefer‐Powell, & Carr, 2000). Processed meats high in sodium are indeed likely to be drivers of CVD, whereas evidence correlating fresh red meat consumption with heart disease is more lacking (McNeill & Van Elswyk, 2012; Micha, Wallace, & Mozaffarian, 2010). Furthermore, when ruminant animals are finished on grass and clovers, their meat tends to have lower quantities of C16:0, higher quantities of C18:0, and ω‐6:ω‐3 ratios of 2:1 or lower (Warren et al., 2008a), likely resulting in reduced risks of CVD and other inflammatory‐driven diseases when consumed in moderation (Simopoulos, 2006).

Such considerable differences in health implications between meat products produced under different feeding regimes make omega‐3 content of meat an ideal case to investigate the effect of accounting for human nutritional aspects of agricultural production systems in the environmental assessment framework. Motivated by this observation, the remainder of the article is structured as a combination of a brief summary of state‐of‐the‐art meat quality research and a two‐part quantitative case study. Following the literature review, the first part of the case study explores the method to simultaneously quantify the impacts of farming systems on resultant fatty acid profiles and accompanied environmental footprints. In the second part, this method is further expanded to incorporate the concept of nutrient indices, with the aim to cover a more diverse range of nutrients both beneficial and detrimental to human health and, by extension, make the approach more holistic. The article concludes with discussions on practical barriers facing the proposed approach and pathways to overcome these challenges.

## 2 EFFECTS OF FARMING SYSTEMS ON MEAT QUALITY

As discussed, it is increasingly recognized that mass‐based assessments of agrifood systems are often inadequate at capturing the complexities of both food production (Martínez‐Blanco, Antón, Rieradevall, Castellari, & Muñoz, 2011) and wider supply chains (Schau & Fet, 2008) and, as a result, nutrition is rapidly becoming a key aspect of food LCA studies (Nemecek, Jungbluth, i Canals, & Schenck, 2016). Sonesson et al. (2017) offer important insight that the shift to quality‐based functional units can dramatically alter the resultant environmental footprints of human diets, although efforts so far have mostly been confined to diet‐level analyses. As will become clear, however, it is possible to extend the concept of nutritional LCA to single‐commodity setting and draw implications on on‐farm practices if the impacts of varied production systems on food quality are systematically elucidated. Using fatty acid profiles of meat products as an example, this section summarizes the current state of knowledge concerning how farm management affects the nutritional value of the final product.

Among various classes of fatty acids, omega‐3 polyunsaturated fatty acids (PUFA) are known to have various health benefits, such as prevention of CVD and rheumatoid arthritis, as well as improvements to brain function and mental stability (Ruxton, Reed, Simpson, & Millington, 2004). While omega‐3 has traditionally been considered beneficial only when maintained in a suitable ratio with omega‐6, some research has subsequently challenged this theory, suggesting that the benefit of omega‐3 should be considered solely in terms of total intake (Stanley et al., 2007). Importantly, omega‐3 content of meat products is known to be manipulated through livestock feeding strategies (Dewhurst, Shingfield, Lee, & Scollan, 2006; McAfee et al., 2010); in other words, a change in on‐farm practice will likely have direct impacts on LCA results when the functional unit is altered from mass‐based to nutrition‐based.

To date, several reviews of the literature have been conducted on the relationship between farming systems and meat quality across different livestock species. Here, by means of systematic selection, nine such articles have been compiled. For the purpose of initial screening, papers containing the keywords “meat quality”, “diet”, and “review” were requested on *Scopus* without any restriction on their publication years. Resulting documents were then sorted according to relevance and the first 200 papers were considered for inclusion. From this pool, all abstracts were examined and studies reporting the effect of either diets or production systems on meat fatty acid profiles were shortlisted. Papers focused solely on novel and unconventional feeding strategies such as inclusion of tannins (Morales & Ungerfeld, 2015) or microalgae (Madeira et al., 2017) were excluded. Furthermore, selection was limited to beef, lamb, chicken, and pork—the four most commonly produced meats globally (OECD/FAO, 2017)—and therefore work on other meat (e.g., rabbit) was also excluded. Of the nine papers selected, the first five primarily review works on white meat, while the last four cover red meat.

D'Arrigo et al. (2011) reviewed a range of fresh and processed meat products with an aim to identify functional foods, or foods which not only provide basic nutrition but also risk prevention from certain types of noncommunicable diseases. The authors acknowledge that improving omega‐3 compositions in the human diet is one of the main premises behind the functional food paradigm, with the adjustment of livestock feed being a key area of potential. For example, Enser, Hallett, Hewitt, Fursey, and Wood (1996) compared fatty acid profiles of beef, lamb, and pork purchased from English retailers. Although pork had the highest PUFA:SFA ratio among the three products due to high levels of C18:2 omega‐6 (linoleic acid), this also resulted in an undesirably high ω‐6:ω‐3 ratio of 7; whereas, the corresponding ratios for beef and lamb were 2 and 1, respectively. While chicken meat was not analyzed as part of this study, its value has subsequently been shown to be comparable (7.6) to that of pork (Lee, Tweed, Kim, & Scollan, 2012).

In a review on meat quality, Wood et al. (2004) summarized possible methods to increase omega‐3 across pork, beef, and lamb systems, e.g., through dietary supplementation using linseed. Supplementation for pigs has shown varying responses, with some studies reporting no adverse effects on meat composition (Enser, Richardson, Wood, Gill, & Sheard, 2000) while others suggesting that feeding strategies which elevate C18:3 (α‐linolenic acid) reduce palatability, particularly when interventional treatments such as salt injection are carried out (Myer et al., 1992).

Employing a systematic review approach, Corino, Rossi, Cannata, and Ratti (2014) examined the effect of dietary linseed on the nutritional quality of pork and pork products. The authors considered the fatty acid profiles of 1006 pigs reported in 24 published papers and found positive effects of linseed supplementation to intramuscular fat and adipose tissue. In addition, a positive correlation between dietary treatment and both α‐linolenic acid and C20:5 (eicosapentaenoic acid; EPA) was noted. While the evidence suggests such supplementation to be largely beneficial, not least due to economic feasibility, the authors highlight an increased risk of rancidity due to the greater oxidation potential of elevated PUFA levels in the meat. As a way to address this issue, they showed that feeding the entire linseed, rather than oil extracts, could decrease oxidation rates and consequently improve the shelf‐life, due to the high levels of antioxidants present in seeds.

Bogosavljević‐Bošković, Rakonjac, Dosković, and Petrović (2012) carried out a review of broiler rearing systems to investigate if production practices affected meat characteristics, such as chemical composition of the end product. Although chicken meat has been shown to be a good source of omega‐3 for humans (Sioen et al., 2006), Bogosavljević‐Bošković et al. (2012) point out that there are conflicting viewpoints on the determining factors of chicken meat quality. For instance, Holcman, Vadnjal, Žlender, and Stibilj (2003) found that chicken meat produced from both indoor and outdoor EU‐regulated fattening operations did not result in significantly different chemical compositions. In contrast, Husak (2008) found that organically reared chickens had higher levels of omega‐3 than meat from free‐range or conventional birds. Unfortunately, these products were obtained from either retailers or wholesalers, and, consequently, their feed ingredients were unknown. Ponte et al. (2004) used controlled trials to examine the effects of alfalfa supplementation on chicken meat. The authors found that, while the legumes improved meat quality, poultry demonstrated lower feed conversion ratios and reduced weight gain, suggesting that forages may not be an efficient feed source for broilers. A later study demonstrated, however, that this negative effect can be partially offset by providing exogenous enzymes to utilize fiber and nonstructural polysaccharides (Lee et al., 2016).

Motivated by declining fish consumption trends in the UK, Rymer and Givens (2005) explored existing literature to determine how omega‐3 fatty acids could be enriched in the human diet via poultry meat. The authors acknowledge that, while typical poultry diets produce meat low in omega‐3 fatty acids, alternative diets enhanced with α‐linolenic acid (typically sourced from linseed) or EPA and C22:6 (docosahexaenoic acid; DHA; typically sourced from marine products) generally result in meat richer in long chain PUFA. Regarding different cuts of meat, dark chicken meat tends to be higher in α‐linolenic acid than white meat, whereas the reverse is true for EPA and DHA due to higher levels of phospholipid fractions in white meat. Nevertheless, the authors point out that the typically low levels of total lipids in white meat result in comparable levels of EPA and DHA across both cuts of meats, and therefore chicken meat, white or brown, could be used as a vehicle to improve uptake of omega‐3 in human diets. As Bogosavljević‐Bošković et al. (2012) noted, however, increased levels of PUFA in meat reduce oxidative stability and consequently shorten shelf‐life unless animals are adequately supplemented with dietary antioxidants such as vitamin E.

Although the conversion efficiency of dietary PUFA into meat is lower for ruminants than for monogastric animals due to biohydrogenation in the rumen (a rumen bacterial response to detoxify unsaturated fatty acid through saturation), basal diets for beef and lamb systems generally contain higher levels of omega‐3; forage, the major component of a ruminant's diet, typically comprises 50%–75% omega‐3 (α‐linolenic acid) and 6%–20% omega‐6 (linoleic acid; Dewhurst et al., 2003). In a review of fatty acid profiles of meat products, Wood et al. (2008) summarized results by Warren et al. (2008a), an examination of the effects of breed (Aberdeen Angus × Holstein‐Friesian vs. Holstein‐Friesian) and diet (grass silage vs. concentrates) on meat quality. The authors found that Holstein‐Friesian steers had higher levels of PUFA and PUFA:SFA ratios than Aberdeen Angus steers because of higher proportions of phospholipids in the total lipids. Grass silage universally increased omega‐3 in the meat, with concentrates conversely increasing omega‐6. However, silage‐fed animals had a lower PUFA:SFA ratio than concentrate‐fed animals, due largely to higher fat deposition. Warren et al. (2008a) also found that as finishing age increased from 14 to 24 months, intramuscular fat levels increased, especially in grass‐silage diets. As with pigs and poultry, increased PUFA had a negative effect on oxidative stability and shelf‐life; as Warren et al. (2008b) reported, however, forage contains high levels of natural antioxidants (carotene and vitamin E) which can inhibit this negative effect.

Reviews by both Scollan et al. (2006) and Howes, Bekhit, Burritt, and Campbell (2015) further explored nutritional strategies to enhance long chain PUFA in beef. Specifically, Scollan et al. (2006) considered the role of genetics in fatty acid composition of meat, such as the thyroglobulin gene that regulates fat marbling and mutations of myostatin that decrease intramuscular fat content and increase muscle mass at the same time. Motivated by health‐conscious consumers, Howes et al. (2015) reviewed current literature to identify opportunities to enhance long chain PUFA in lamb fattening systems. Notably, the authors considered how specific cultivars of herbs and legumes might affect fatty acid profiles; Ådnøy et al. (2005), for example, demonstrated that botanically diverse mountainous swards (classified as native mixed pastures) produced lamb meat with higher levels of PUFA than lowland lamb. Howes et al. (2015) hypothesized that such increases to PUFA could result from a decrease in biohydrogenation caused by endogenous plant factors of a diverse sward. Factors contributing to reduced biohydrogenation were separately reviewed by Lee (2014) and Buccioni, Decandia, Minieri, Molle, and Cabiddu (2012); as an example, red clover (*Trifolium pratense*) facilitates the flow of PUFA to the duodenum and then deposition into meat and milk, through the action of the enzyme system polyphenol oxidase in the rumen.

Venkata Reddy et al. (2015) carried out a review of papers studying differences in meat quality between animal sexes (e.g., heifers and steers). The authors highlight that the hormonal status of cattle plays a significant role in fat and protein distribution within muscles. For example, and perhaps unsurprisingly, they assert that meat quality from heifers is much higher than bulls, largely due to increased fat deposition in heifers which results in improved water‐holding capacity. Consistent with the finding by Ardiyanti et al. (2009) that allele C in heifers produced higher levels of monounsaturated fatty acids (MUFA) and PUFA (as well as lower levels of SFA), consumer panels have also demonstrated a preference for heifer beef over steer beef. More generally, feeding strategies that influence fatty acid profiles have implications on flavor and, consequently, preference; this point was exemplified by Sañudo et al. (2000), when British (grass‐fed) and Spanish (concentrate‐fed) lamb were offered to sensory panels in both countries. The panel in Britain preferred grass‐fed lamb, whereas the Spanish panel preferred concentrate‐fed lamb, reporting distaste for the “grassy” flavor. A similar tendency was observed by Larick and Turner (1990) for US sensory panels, who also preferred concentrate‐fed beef over pasture‐fed beef. Collectively, these results demonstrate that familiarity is a driving force behind consumers’ decision making.

## 3 MATERIALS AND METHODS

### 3.1 Omega‐3 case study

In order to accurately connect the nutritional quality of meat products outlined above to the environmental footprints of farming systems under which they are produced, the following four steps need to be considered along the supply chain: (a) the environmental footprint per unit of farm‐gate output (liveweight) under the studied system; (b) kill‐out percentage of that particular animal; (c) meat yield from the carcass of that particular animal, and; (d) the nutrient content of meat from that particular animal. For the present case study, two functional units were selected based on the method of a preceding study (Marshall, 2001), namely the total mass of omega‐3 PUFA and the combined mass of EPA and DHA, which together constitute a subgroup of omega‐3 that are significantly more biologically active than shorter chain omega‐3 and, therefore, do not need to compete with omega‐6 for desaturase and elongase enzymes. The environmental footprints of different farming systems were estimated by combining studies that collectively cover the above four steps. Seven “treatments” or combinations of species and production systems commonly observed in the UK were identified: intensive cattle, extensive cattle, upland lamb, lowland lamb, conventional chicken, free‐range chicken, and conventional pork. Feeding strategies reflected typical production practices for each system and therefore did not include supplementation of omega‐3‐rich feeds such as linseed. For each treatment, an LCA study and a meat science study reporting the fatty acid profiles were matched as closely as possible with respect to the underlying farming systems (Table 1), and the global warming potential (GWP) was derived under each functional unit. GWP based on a standard mass‐based functional unit (kg deboned meat) is also reported for methodological comparison.

**Table 1:** Unit comparability between preceding works selected for the case study

Species	System	GWP study	GWP unit	Carcass study	Carcass unit	Omega‐3 study	Omega‐3 unit (mg/100 g meat)
Beef	Concentrate	Audsley and Wilkinson (2014)	7.9 kg CO_2_‐eq/kg CW	van Leeuwen (2014a)	0.87 kg meat/kg CW	Warren et al. (2008a)	20
	Forage		15.9 kg CO_2_‐eq/kg CW				97
Lamb	Lowland	Jones et al. (2014)	10.9 kg CO_2_‐eq/kg LW	van Leeuwen (2014b)	0.88 kg meat/kg CW^a^	Whittington et al. (2006)	94
	Upland		12.9 kg CO_2_‐eq/kg LW				103
Chicken	Intensive	Leinonen et al. (2012)	4.4 kg CO_2_‐eq/kg MW	Leinonen et al. (2012)	Not required	Givens et al. (2011)	362
	Free range		5.1 kg CO_2_‐eq/kg MW				214
Pork	Intensive	Audsley and Wilkinson (2014)	4.0 kg CO_2_‐eq/kg CW	Marcoux et al. (2007)	0.54 kg meat/kg CW	Enser et al. (1996)	51

*Notes*

CW: carcass weight; LW: liveweight; MW: meat weight.

a Converted from LW based on the kill‐out rate estimated by van Leeuwen (2014b).

Data pertaining to beef‐related emissions were sourced from Audsley and Wilkinson (2014), of which dairy beef systems (slaughtered at 13 months) and suckler beef systems (18–19 months) were judged to be the most comparable, respectively, to the concentrate‐fed beef and the silage‐fed beef examined in Warren et al. (2008a). For fatty acid profiles, data from Holstein‐Friesian cattle (on two feeding regimes) slaughtered at 19 months were adopted. As Audsley and Wilkinson (2014) utilize carcase weight as a functional unit, meat yield was estimated using the guidelines by van Leeuwen (2014a), which suggest the combined wastage rate (bone/fat/drip loss) of 13.0%.

Lamb production in the UK is typically carried out on both lowland and upland. To examine differences arising from these contrasting production environments, carbon footprints associated with both systems were sourced from Jones, Jones, and Cross (2014). As the functional unit adopted by the authors was 1 kg liveweight, GWP was first converted to represent 1 kg carcase weight (using the kill‐out coefficient of 47.4%) and then to 1 kg edible meat (using the combined wastage rate of 12.2%), both based on van Leeuwen (2014b). Fatty acid profiles were sourced from Whittington, Dunn, Nute, Richardson, and Wood (2006), who conducted meat analysis of Suffolk lambs produced under lowland and upland systems.

Global warming potential arising from broiler production was obtained from Leinonen, Williams, Wiseman, Guy, and Kyriazakis (2012), which employed a functional unit of expected weight of edible meat. As a result, no manipulations were made to derive the meat yield. Fatty acid composition was taken from Givens, Gibbs, Rymer, and Brown (2011), who used whole cooked chickens for their meat analysis. Although cooked meat could potentially lose a portion of PUFA content as a consequence of oxidation, recent research has demonstrated that these losses are likely to be minimal (Douny et al., 2015).

LCA data for pork production was sourced from Audsley and Wilkinson (2014), whose study of typical pig production systems in the UK was carried out with the functional unit based on carcass weight. Meat yield was obtained from Marcoux, Pomar, Faucitano, and Brodeur (2007), whereby 53.9% of a carcass is reported to be lean meat. Meat data were taken from Enser et al. (1996), who examined the fatty acid composition of typical pork cuts available at UK retailers. As the feeding regime of the animals used in the meat analysis was unknown, it was assumed that the cuts represent conventional (intensive) farming systems.

### 3.2 Nutrient index case study

While the above approach offers a useful framework for LCA when the research question primarily concerns a single nutrient, these functional units do not necessarily represent the overall value of the product associated with human nutrition. One way to address this issue is through the use of a nutrient index, a scalar value to combine information on multiple nutrients, both beneficial and detrimental to human health. For the present analysis, four variants of the formulae originally developed by Saarinen, Fogelholm, Tahvonen, and Kurppa (2017) for protein‐rich foods in Finland were adopted and applied to the same seven livestock systems as above: *UKNI*_*prot*_ *7* and *UKNI*_*prot*_ *10* based on *FNI*_*prot*_ *7*, and *UKNI*_*prot*_ *7‐2* and *UKNI*_*prot*_ *10‐2* based on *FNI*_*prot*_ *7‐2*. The first group simply rewards foodstuffs with higher contents of desirable nutrients (protein, MUFA, EPA + DHA, calcium, iron, riboflavin, folate and, additionally for *UKNI*_*prot*_ *10*, vitamin B12, selenium, zinc), while the second group also penalizes those with higher contents of undesirable nutrients (SFA and sodium). Only EPA and DHA were considered among PUFA, so as to ensure their bioavailability. Vitamin B12, selenium, and zinc, which did not form part of the original indices, were added to the alternative specifications as meat is particularly rich in these micronutrients (Castañé & Antón, 2017). All four indices are expressed as % RDI per 100 g, indicating the proportion of RDI satisfied across all nutrients, minus penalty where applicable, by the said amount of product.

RDI and RDA values were sourced from the British Nutrition Foundation (BNF, 2016) as averages between female and male. Where UK‐specific recommendations were unknown or unspecified, as was the case with MUFA, values from Saarinen et al. (2017) were directly adopted. Nutritional compositions of (uncooked) meats were sourced from McCance and Widdowson (2015), except for fatty acid profiles carried over from the first case study (Table 1). GWP estimates were also taken from the first case study and, contrary to Saarinen et al. (2017), excluded the cooking process to match nutritional data. Based on best available evidence, it was assumed that protein and micronutrient contents were comparable between production systems for the same species (Scollan et al., 2006).

## 4 RESULTS

### 4.1 Omega‐3 case study

Table 2 provides a breakdown of fatty acid profiles adopted for the seven treatments. Mirroring the results from other studies, considerable differences were found between animals fed concentrates and forages, with more extensive systems generally producing more favorable profiles. Interestingly, omega‐3 content of free‐range chickens was found to be lower than that of conventionally reared chickens. For many consumers who believe that free‐range or organic meat products are healthier (Van Loo et al., 2010), this result may be unexpected. However, since the study (Givens et al., 2011) was based on meat purchased from supermarkets, the diets of chickens are unknown and, as a consequence, the reasons behind the PUFA differences cannot be completely ascertained.

**Table 2:** Summary of omega‐3 and 6 fatty acid profiles reported in preceding works selected for the case study

Species	System	Study	Omega‐3 (mg/100 g meat)	DHA + EPA (mg/100 g meat)	ω‐6:ω‐3
Beef	Concentrate	Warren et al. (2008a)	20.3	3.4	14.4
	Forage		97.2	27.4	1.2
Lamb	Lowland	Whittington et al. (2006)	94.0	26.4	1.2
	Upland		103	31.7	1.5
Chicken	Intensive	Givens et al. (2011)	362	17.6	5.5
	Free range		214	14.7	7.6
Pork	Intensive	Enser et al. (1996)	51.3	14.8	7.4

*Notes*

DHA and EPA are a subgroup of omega‐3 fatty acids that are the most biologically active and do not need to compete with omega‐6 for enzymes.

DHA: docosahexaenoic acid; EPA: eicosapentaenoic acid; ω‐6:ω‐3: the mass ratio between omega‐6 and omega‐3 fatty acids.

Global warming potential implications derived under the new functional units were profoundly different compared to the standard LCA results, particularly for beef and sheep systems (Table 3). For example, concentrate‐fed cattle produced approximately half the emissions of pasture‐fed cattle under the standard mass‐based approach. When omega‐3 content of meat is considered, however, these results reversed and the concentrate‐based system produced more than double the emissions of the pasture‐based beef system. This difference was further exacerbated when only the most bioactive omega‐3 fatty acids (EPA and DHA) were included. Between the two lamb systems, while the upland system had a marginally higher GWP, it also produced meat with a marginally higher omega‐3 content, resulting in a minimal difference when the novel functional units were applied. Differences between free‐range and broiler chickens were less pronounced because neither GWP nor omega‐3 contents differ as substantially as cattle and lamb systems. Nonetheless, the higher levels of total omega‐3 and EPA + DHA contained in intensively reared chickens increased the GWP gap between the two systems.

**Table 3:** Global warming potential (GWP) under different functional units

Species	System	Mass‐based GWP (kg CO_2_‐eq/kg meat)	Quality‐based GWP (kg CO_2_‐eq/g omega‐3)	Quality‐based GWP (kg CO_2_‐eq/g EPA + DHA)
Beef	Concentrate	9.8^a^	48.0	288.1
	Forage	18.3^a^	18.5	67.7
Lamb	Lowland	26.1^a^	28.7	99.2
	Upland	30.9^a^	30.0	98.9
Chicken	Intensive	4.4	1.2	25.1
	Free range	5.1	2.4	34.7
Pork	Intensive	7.4^a^	14.4	50.3

*Notes*

DHA and EPA are a subgroup of omega‐3 fatty acids that are the most biologically active and do not need to compete with omega‐6 for enzymes.

DHA: docosahexaenoic acid; EPA: eicosapentaenoic acid.

a Recalculated from values reported by the authors for cross‐study comparability.

Across species, pig production was shown to be most affected when the functional unit was changed from mass‐based to quality‐based. While the new method did not alter the relative rankings between species, the discrepancy between red meat systems and white meat systems was considerably narrowed, challenging the view to stringently regulate ruminant production on the basis that it is far more harmful to society than monogastric production (Springmann et al., 2017). It could be argued that omega‐3 should be sourced from alternative food groups such as oily fish and seafood, which are generally known to have higher contents of EPA and DHA than either white meat or red meat. Nonetheless, low consumption of these items in many societies suggests that, at least in short to medium terms, it is important to evaluate environmental impacts associated with production of all food types based on their nutritional values. More importantly, the current approach could be applied to any number of nutrients, so as to draw information not reflected when the mass of product is used as a sole reference to the value of food.

Finally, it is worthwhile reiterating that, in addition to containing higher levels of omega‐3, forage‐based production systems are also associated with lower ω‐6:ω‐3 ratios (Table 2). Although quantifying this effect within the LCA framework is not straightforward, these systems are likely to result in further health benefits for humans than what is shown under the proposed functional units.

### 4.2 Nutrient index case study

When the seven systems were compared by the absolute level of nutrient scores, beef produced from forage‐fed cattle was shown to be the most favorable product under all four index specifications (Table 4). All other systems, apart from intensive pork, performed comparably under *UKNI*_*prot*_ *7*, with pork scoring low due to lower contents of protein, MUFA and folate. Under *UKNI*_*prot*_ *7‐2* that also considers the two nutrients to be limited, beef and lamb produced the highest scores, while pork overtook free‐range chicken due to its low SFA and Na. When the three additional nutrients (vitamin B12, Se, and Zn) were further included (under *UKNI*_*prot*_ *10* and *UKNI*_*prot*_ *10‐2*), both beef production systems became notably more favorable than their counterparts from other species, owing to high concentrations of vitamin B12 and Zn. This finding is notable not only in the comparison between red meat and white meat but also between meat‐based diets and plant‐based diets, as vegan diets are often deficient in B12 and Zn, the latter more so among children (Gibson, 1994; Pawlak et al., 2013).

**Table 4:** Nutritional composition of each meat product (100 g) considered

Nutrient/index	Unit	RDI/RDA^a^	Beef		Lamb		Chicken		Pork
			Concentrate	Forage	Lowland	Upland	Intensive	Free range	Intensive
Protein	g/day	50.25	23.5	23.5	20	20	26.3	26.3	18.6
MUFA	g/day	37.5	1.1	1.6	1.3	1.1	3.7	5.4	0.9
EPA+DHA	mg/day	250	3.4	27.4	26.4	31.7	17.6	14.7	14.8
Ca	mg/day	700	5	5	12	12	11	11	10
Fe	mg/day	11.75	1.6	1.6	1.4	1.4	0.7	0.7	0.4
Riboflavin	mg/day	1.2	0.26	0.26	0.2	0.2	0.15	0.15	0.18
Folate	μg/day	200	16	16	6	6	9	9	1
Vitamin B12	μg/day	1.5	2	2	1	1	0	0	1
Se	μg/day	67.5	8	8	3	3	15	15	11
Zn	mg/day	8.25	4	4	2	2	1.5	1.5	1.3
Na^b^	g/day	6	0.07	0.07	0.07	0.07	0.08	0.08	0.053
SFA^b^	g/day	25	1.1	1.5	1.3	1.2	2.4	3.7	0.9
*UKNIprot7*	% RDI		13.6	15.2	12.4	12.7	13.4	13.9	9.4
*UKNIprot7‐2*	% RDI		10.7	11.6	9.2	9.7	7.9	5.9	7.1
*UKNIprot10*	% RDI		28.9	30.0	18.2	18.4	13.4	13.8	16.4
*UKNIprot10‐2*	% RDI		26.0	26.4	15.0	15.4	7.9	5.7	14.2

^a^Recommended daily intake/allowance based on BNF (2016) and Saarinen et al. (2017). ^b^Nutrients to be discouraged.

**Figure 1:**
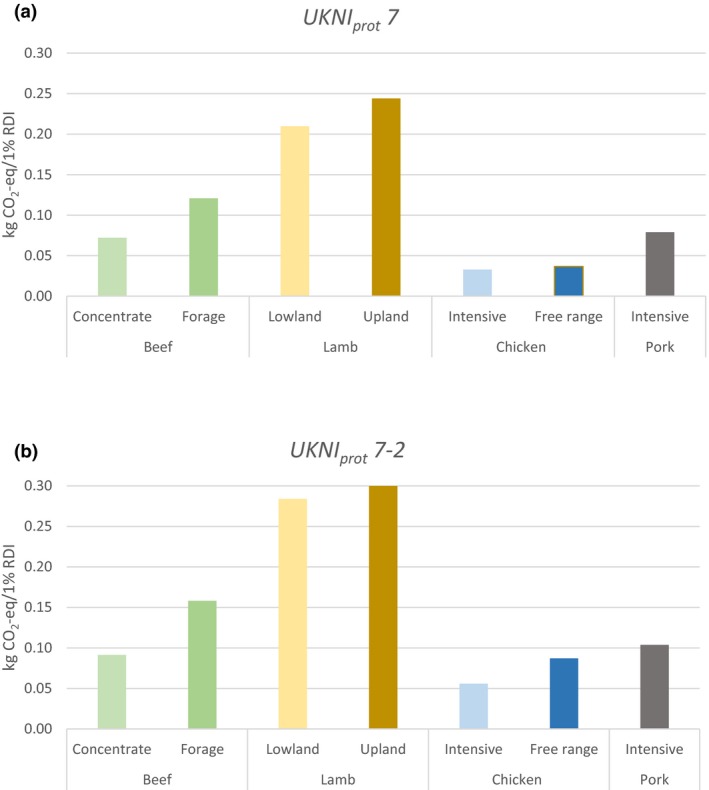
Global warming potential scaled to 1% of RDI under (a) *UKNI*_*prot*_ *7* and (b) *UKNI*_*prot*_ *7‐2* specifications

**Figure 2:**
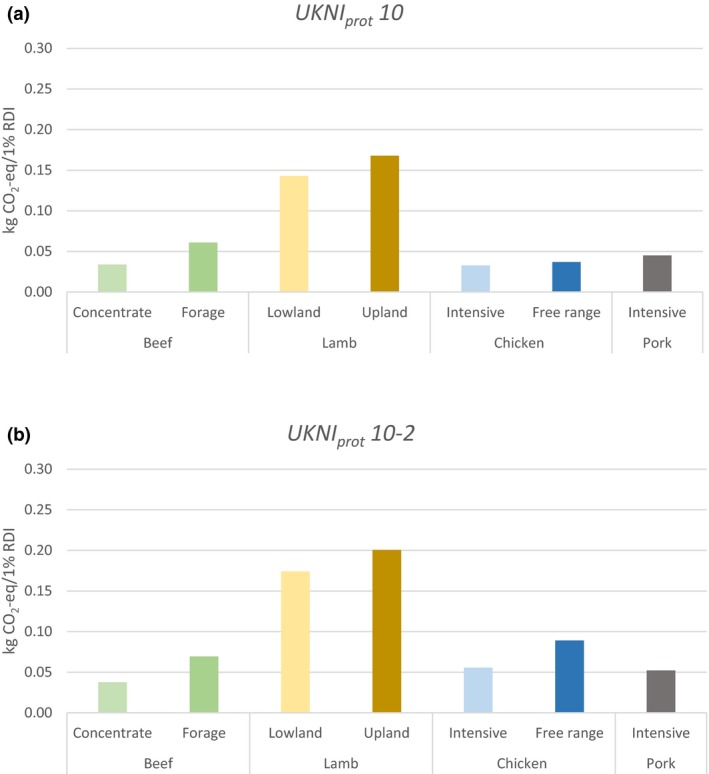
Global warming potential scaled to 1% of RDI under (a) *UKNI*_*prot*_ *10* and (b) *UKNI*_*prot*_ *10‐2* specifications

For computation of GWP, the mass‐based functional unit (Table 3) was replaced with the four nutrient indices as denominators. As all nutrient scores are expressed as percentage, GWP values represent the environmental burdens associated with 1% of an average British person's nutrient intake in the form of that particular meat. It was found that the low mass‐based GWP of chicken systems directly translated to low environmental impacts under both *UKNI*_*prot*_ *7* and *UKNI*_*prot*_ *7‐2* (Figure 1). The largely positive nutritional profiles of beef and, to a lesser extent, lamb, did not greatly alter the relative rankings under these index specifications. However, when vitamin B12, Se, and Zn were introduced as nutrients to be encouraged, notable reversals in rankings were observed for cattle systems (Figure 2). Concentrate beef generated the second lowest GWP only after intensive chicken under *UKNI*_*prot*_ *10*, and the lowest under *UKNI*_*prot*_ *10‐2*. The performance of forage beef also improved, producing lower emissions than free‐range chicken under *UKNI*_*prot*_ *10‐2*. On the other hand, lamb systems consistently generated the highest burdens regardless of the index specifications, due to the significantly high mass‐based GWP that were robust to different functional units. Nonetheless, the overall findings of this analysis question the appropriateness of comparing environmental performances of products on a mass basis—in a similar vein to the first case study.

## 5 DISCUSSION

While recent studies investigating the environmental impacts of alternative diets provide useful framework for assessing implications of different food consumption patterns on the whole, the LCA literature remains short of methodologies to account for quality differences between individual foodstuffs produced under contrasting on‐farm practices. The results from the above case studies suggest that the application of nutrition‐based functional units in the single‐commodity setting has the potential to fill this research gap and offer better insight into economic‐environmental trade‐offs inherent by each production system and, by extension, on‐farm practices that should be promoted. Relative environmental performances among different agricultural systems reversed as new functional units were adopted, in particular between pasture‐based and concentrate‐based livestock systems, highlighting that the effect of farming methods on product quality should not be ignored in comparative studies. Nevertheless, improving nutritional values of meat (per GHG emissions) is only beneficial to the environment if it is accompanied by improved consumer awareness of differences in food quality (Coelho et al., 2016), which subsequently leads to reduction in consumption of lower quality products. To this end, there is a clear need for further interdisciplinary work, including a scope for consequential LCA to account for wider socioeconomic impacts of dietary transitions as well as for endpoint LCA to consider the ultimate impact of a product (and its quality) on human health. Even though a greater degree of uncertainty makes the latter a challenging task, work carried out by Stylianou et al. (2016), whereby endpoint impacts on health and environmental were concomitantly quantified, has paved the way to implement this concept. Finally, it should also be noted that GWP is one of many aspects of sustainability (Takahashi et al., 2018); in order to achieve a truly holistic comparison of livestock systems, a suite of metrics should collectively be considered, including those representing animal welfare (Edgar, Mullan, Pritchard, McFarlane, & Main, 2013), land use (Wilkinson & Lee, 2017), and water quality (Leip et al., 2015), to name a few.

Needless to say, the validity of the proposed approach depends upon data reliability and the relative importance of the nutrients incorporated into the analysis. As already discussed, information from four steps along the supply chain (production, slaughtering, packing, and consumption) needs to be linked together to enable the proposed framework and, while they should ideally be collected from a single agricultural system within a single region, such opportunities are rare and far between. The alternative method of collating separate works together poses the risk of inappropriately linked parameters, as carcass conformation, meat yield, chemical composition of meat and ultimately its human nutritional value are all strongly influenced by farming strategies that fundamentally regulate flow of nutrients—from soil to crops and then to animals.

As demonstrated here, it is possible to utilize existing datasets (from unrelated experiments carried out under similar environments) and create “hypothetical supply chains” that are sufficiently realistic for exploratory purposes. However, as the degree of uncertainty surrounding this approach cannot be specified, ideally a better way forward to overcome this issue would be to employ a whole supply chain approach (Orr et al., 2016), whereby actual products originating from different on‐farm treatments are marked and tracked along the marketing process and used for quality evaluation and consumer trials. The finding from the present study, namely that nutritional quality rather than quantity is likely to play a key role in sustainable livestock production, warrants future studies in this area.

## CONFLICT OF INTEREST

None declared.
